# Impact of the highly active antiretroviral therapy era on the epidemiology of primary HIV-associated thrombocytopenia

**DOI:** 10.1186/s13104-015-1548-3

**Published:** 2015-10-23

**Authors:** Thomas A. O’Bryan, Jason F. Okulicz, William P. Bradley, Anuradha Ganesan, Xun Wang, Brian K. Agan

**Affiliations:** Infectious Disease Clinical Research Program, Uniformed Services University of the Health Sciences, Bethesda, MD USA; Henry M Jackson Foundation for the Advancement of Military Medicine, Bethesda, MD USA; Infectious Disease Service, San Antonio Military Medical Center, 3551 Roger Brooke Drive, Fort Sam Houston, TX 78258 USA; Walter Reed National Military Medical Center, Bethesda, MD USA

**Keywords:** HIV, Primary, Thrombocytopenia, Antiretroviral, HAART, Incidence, Viremia, CD4

## Abstract

**Background:**

Primary HIV-associated thrombocytopenia (PHAT) typically improves with highly active antiretroviral therapy (HAART); however, cases continue to occur. Data comparing the epidemiology of PHAT between the pre-HAART and HAART eras are limited. We retrospectively examined the incidence of PHAT over 28 years in the US Military HIV Natural History Study (NHS) from 1986 to 2013.

**Results:**

Subjects had a nadir platelet count <100 × 10^9^/l with no other identifiable cause. Time periods were categorized as pre-HAART (1986–1995), early HAART (1996–2001), and later HAART (2002–2013). Incidence, demographic data, and CD4 count were compared across the three eras. A generalized estimating equations model was used to assess any association of platelet count and HIV viral load in cases diagnosed during the HAART eras. 218 participants met the case definition. 86.2 % of cases occurred prior to 2002. The incidence of PHAT per 1000 person-years of follow-up was 16.3, 4.6, and 1.9 during pre-HAART, early HAART and later HAART eras respectively. CD4 cell counts were significantly higher in the HAART eras at the time of thrombocytopenia (p < 0.001). Of patients diagnosed after 1996, 96.4 % were viremic within six months preceding the platelet nadir and over half were antiretroviral naïve. Viral load (per log_10_ copies/ml) inversely correlated with platelet count throughout the HAART eras (p < 0.0001).

**Conclusions:**

The incidence of PHAT has markedly decreased in the HAART era. However, viremic individuals, including those with healthy CD4 cell counts, may be at risk. Achieving viral suppression as early as possible may decrease the incidence further.

## Research hypothesis

The incidence and characteristics of primary HIV-associated thrombocytopenia have changed in the era of highly active antiretroviral therapy.

## Background

Thrombocytopenia, in the absence of secondary causes (e.g. drugs, opportunistic infections, malignancy), has been observed in HIV-infected persons since the beginning of the epidemic [1]. Primary HIV-associated thrombocytopenia (PHAT) may occur at any stage of HIV disease. In the early stages of infection, the clinical picture may be identical to immune thrombocytopenia purpura (ITP) with accelerated platelet destruction related to immune complexes and cross-reacting platelet antibodies [2]. As HIV disease progresses, thrombocytopenia may develop by other virus-related mechanisms including suppression of platelet replication, shortening of platelet life span, and direct destruction of megakaryocytes [3, 4].

PHAT typically improves with highly active antiretroviral therapy (HAART) [5–7]. However, cases continue to be observed. Although it is generally presumed that the incidence declined from the pre-HAART era, data comparing the pre-HAART and HAART eras are limited [8]. Understanding the effect of HAART on the epidemiology of PHAT may have implications regarding the timing of HAART initiation. In this study, we examined the change of incidence and characteristics of PHAT from the pre-HAART to HAART era in a US military population from 1986 to 2013. We also examined the relationship of thrombocytopenia with HIV viral load and CD4 count.

## Methods

The US Military HIV Natural History Study (NHS) is an ongoing, continuous enrollment observational cohort of Department of Defense active duty and beneficiaries diagnosed with HIV infection, followed at six military medical centers in the United States and has been previously described [9]. Enrolling since 1986, the NHS has approximately 5700 participants with signed written consent. Approval for this research was obtained by the Ethics Committees of the Uniformed Services University of the Health Sciences, Bethesda, MD and each participating site (San Antonio Military Medical Center, Fort Sam Houston, TX, Walter Reed National Military Medical Center, Bethesda, MD, Naval Medical Center Portsmouth, Portsmouth, VA, Naval Medical Center San Diego, San Diego, CA, Tripler Army Medical Center, Honolulu, HI, and Madigan Army Medical Center, Tacoma, WA).

Following enrollment, subjects have study visits approximately every 6 months. Data collected at each visit include demographic information, past and interim medical histories and illnesses, medications, vaccinations, and standard clinical laboratory studies, including annual screening for hepatitis B and C viruses. Due to prior Department of Defense policy (“Don’t Ask, Don’t Tell”), HIV exposure category has not been routinely captured for the majority of the study visit. However, rates of HIV risk behaviors have been previously reported and intravenous drug use is rare (<3 %) [10].

Data from the NHS was retrospectively analyzed from 1986 to 2013. Cases were identified as those subjects who received a diagnostic code for ITP (defined as thrombocytopenia <150 × 10^9^/l on at least two consecutive occasions and a nadir platelet count <100 × 10^9^/l, and no other secondary causes including drugs and opportunistic infection were identified). Subjects initially diagnosed with ITP were excluded if they were coinfected with hepatitis B or C or subsequently found to have cirrhosis, leukemia, or solid tumor malignancy with exception of non-melanoma skin cancer. This definition is consistent with that recommended by an ITP International Working Group of recognized experts [11]. For this study, we elected to use the term “primary HIV-associated thrombocytopenia” in recognition that immune and nonimmune mechanisms may be involved. Variables included demographics (age, gender, race), platelet counts, CD4 cell count and HIV viral load within 6 months preceding the nadir platelet count. Time periods were classified as pre-HAART (1986–1995), early HAART (1996–2001), and later HAART (2002–2013). Person-years of follow-up (PYFU) for each subject was measured as the time from the documented date of seropositivity to the time of last visit or established diagnosis of PHAT, whichever occurred first.

Comparisons were made between the three eras in incidence of primary HIV-associated thrombocytopenia (cases per 1000 person-years of follow-up), age, race, gender, and CD4 cell count and HIV viral load within 6 months preceding the platelet nadir. Descriptive statistics utilized Chi squared test for categorical variables and Kruskal–Wallis test for continuous variables. A generalized estimating equations model was used to assess any association between HIV viral load and platelet count in patients diagnosed with PHAT during the HAART eras.

## Results

5697 subjects enrolled in NHS from 1986 to 2013. Of these, 3231 enrolled during the pre-HAART era (1986–1995), 955 enrolled during the early HAART era (1996–2001), and 1551 enrolled during later HAART era (2002–2013). PYFU during each time period was 9282 (pre-HAART era), 8005 (early HAART era), and 16,150 (later HAART era). 218 participants met the case definition of PHAT (Table 1).

**Table 1:** Characteristics of subjects diagnosed with primary HIV-associated thrombocytopenia in the US Military Natural History Study 1986–2013

	Pre-HAART era 1986–1995	Early HAART era 1996–2001	Later HAART era 2002–2013	P value
No. of cases	151	37	30	
Median age (yrs) (IQR)	32 (27, 38)	34 (31, 39)	36 (29, 41)	NS
Male gender	138 (91.4 %)	36 (97.3 %)	30 (93.3 %)	NS
Race				NS
Caucasian	87 (57.6 %)	25 (67.6 %)	17 (56.7 %)	
African–American	52 (34.4 %)	9 (24.3 %)	10 (33.3 %)	
Hispanic	10 (6.6 %)	2 (5.4 %)	2 (6.7 %)	
Other	2 (1.4 %)	1 (2.7 %)	1 (3.3 %)	
Nadir platelet count				
<50 × 10^9^/l	73 (48.3 %)	11 (29.7 %)	6 (20.0 %)	0.005
<20 × 10^9^/l	33 (21.8 %)	6 (16.2 %)	4 (13.3 %)	NS
CD4 count (cells/μl)				
≤100	59 (39.1 %)	9 (24.3 %)	2 (6.7 %)	
101–200	20 (13.2 %)	6 (16.2 %)	1 (3.3 %)	
201–350	26 (17.2 %)	11 (29.7 %)	10 (33.3 %)	
351–500	19 (12.6 %)	4 (10.8 %)	8 (26.7 %)	
>500	26 (17.2 %)	6 (16.2 %)	9 (30.0 %)	
Not determined	1 (0.7 %)	1 (2.7 %)		
Median (IQR)	156 (30, 406)	262 (148, 378)	380 (255, 517)	<0.001

*HAART* highly active antiretroviral therapy, *IQR* interquartile range

The majority of cases (86.2 %) occurred prior to 2002. The incidence per 1000 PYFU markedly decreased over time with a rate of 16.3, 4.6, and 1.9 during the pre-HAART, early HAART and later HAART eras respectively (p < 0.001). Platelet count was more likely to fall below 50 × 10^9^/l in the pre-HAART era (odds ratio 2.75, 95 % confidence interval 1.46–5.20, p = 0.002). However, the proportion of cases in which the platelet count nadir was less than 20 × 10^9^/l did not significantly differ across the three eras.

CD4 cell count within 6 months prior to the platelet nadir was recorded in 216 participants and progressively shifted to higher values over the three eras. In the pre-HAART era, CD4 cell counts were ≤200 cells/μl in 52.3 % of the thrombocytopenic cases compared to 40.5 and 10 % in the early and later HAART eras respectively. The proportion of subjects with CD4 cell counts above 350 cells/μl were similar in the pre-HAART (29.8 %) and early HAART (27.8 %) eras but was higher (56.7 %) during the later HAART era.

Of the 67 patients who became thrombocytopenic during the HAART eras, 37 (55.2 %) were antiretroviral-naïve. This included 11 of 15 cases where the CD4 count was >500 cells/μl. Laboratory data in which HIV viral load was recorded within 6 months preceding the nadir platelet count was available in 56 subjects. Of these, HIV viremia was detected in 54 (96.4 %) participants, of whom 29 (53.7 %) had not previously received antiretroviral therapy. Median (IQR) viral load prior to the platelet nadir was 32,531 (4473, 78,385) copies/ml.

All platelet counts and HIV viral load assays which were drawn simultaneously from 1996 to 2013 were studied among subjects diagnosed with PHAT at any time during the HAART era. This included 1009 data points in 63 subjects and showed a strong association between time updated viral load per log_10_ copies/ml and platelet count. This relationship was highly significant (p < 0.0001) using a generalized estimating equation model. Each rise in one viral load log_10_ corresponded with a decline of 19 × 10^9^/l platelets.

## Discussion

In our US military cohort, the incidence of PHAT declined approximately eight-fold after 2001 compared to the pre-HAART era. Cases diagnosed during the HAART era were characterized by significantly higher CD4 cell counts. In cases where recent viral load data was available, we found that nearly all cases diagnosed in the HAART era coincided with detectable HIV viremia. Our study is consistent with other reports showing a strong correlation between viral load and platelet count in HIV-associated thrombocytopenic patients [5–7, 12].

Prior to the advent of HAART, thrombocytopenia, defined as platelet count <150,000 × 10^9^/l, was identified in 10–30 % of HIV-infected patients with the incidence increasing with progression of disease [13]. In the HAART era, several studies have found an incidence of 10–15 %, using the same cut-off value [13]. In a recent retrospective study of a large Italian cohort, severe thrombocytopenia (<50 × 10^9^/l) developed in 597 of 5137 subjects from 1985 to 2012, with the incidence declining over time from 63.4/1000 PYFU (1985–1989) to 14.9/1000 PYFU in the HAART era (1997–2012) [14]. Studies of thrombocytopenia in HIV-infected patients vary in case definition, inclusion of secondary causes, coinfections, or focus on clinical features and outcome. For instance, in the Italian cohort, 44.4 % of the subjects were seropositive for hepatitis C virus (HCV), 34 % had chronic liver disease and 14.7 % were diagnosed with malignancy. Thus, only 35 of the reported cases over a 28 year period could be classified as primary HIV-related thrombocytopenia [14].

Because of the unique features of our cohort, we focused our study on cases of thrombocytopenia where HIV infection was the only identifiable predisposing factor. Our subjects have free access to health care and low incidence of intravenous drug abuse. Because of mandatory screening for HIV infection in the US military, most of our subjects enroll in the cohort early in the course of HIV infection, while in relatively good health. Our participants are screened yearly for hepatitis B and C viruses. Because HCV serology was not available prior to 1990, negative antibody testing could not be recorded in 28 of our PHAT cases in the pre-HAART era. These included 23 subjects born between 1945 and 1965, the birth cohort with highest prevalence of HCV infection. Although these persons did not have risk factors for HCV or signs of liver disease, we recognize the possibility that some of these cases may have had undiagnosed HCV co-infection. However, we do not feel this would change our results significantly. Had all 28 subjects been excluded from analysis, the pre-HAART incidence would have dropped from 16.3 to 13.3 cases/1000 PYFU and a seven-fold reduction in the later HAART era.

Although our results may not be representative of other HIV-infected populations, fewer confounding variables found in NHS subjects may allow clearer distinction of cases of primary HIV-associated thrombocytopenia and more accurate analysis of the longitudinal changes in epidemiology associated with the HAART eras. In contrast to thrombocytopenia more commonly present in persons with late stage HIV disease prior to the HAART era, the majority of cases in the later HAART era were associated with CD4 cell counts >350 cells/μl. Furthermore, almost all of the cases diagnosed after 1996 had detectable HIV viremia. These results are similar to a study of 73 HIV-infected thrombocytopenic (<100 × 10^9^/l) patients in New York City where HIV viremia was detected in 21 of 22 subjects without HCV or cirrhosis [15]. More than half of our subjects with thrombocytopenia after 1996 were HAART-naïve, consistent with HAART initiation guidelines at the time based on their CD4 cell levels. Our study is limited by its retrospective nature and under-representation of women. Clinical presentation and treatment of thrombocytopenia were not included in the design.

In conclusion, the HAART era has had a marked effect on the incidence of PHAT, although viremic individuals, including those with healthy CD4 cell counts, may be at risk. Further decline in incidence may be another potential benefit to beginning HAART as early as possible [16].

## Abbreviations

HIV: human immunodeficiency virus
PHAT: primary HIV-associated thrombocytopenia
ITP: immune thrombocytopenic purpura
HAART: highly active antiretroviral therapy
NHS: US Military HIV Natural History Study
PYFU: person-years of follow-up
IQR: interquartile range
